# A Systematic Review of the Attitudes, Beliefs, and Acceptance of the COVID-19 Vaccine in the Western and Eastern Hemispheres

**DOI:** 10.7759/cureus.73161

**Published:** 2024-11-06

**Authors:** FNU Sorath, Sheena Shiwlani, FNU Sindhu, Abhi C Lohana, Yaqub Nadeem Mohammed, Subhash Chander, Roopa Kumari

**Affiliations:** 1 Anesthesia, Dow University of Health Sciences, Karachi, PAK; 2 Internal Medicine, Mount Sinai Hospital, New York, USA; 3 Internal Medicine, Jinnah Postgraduate Medical Centre, Karachi, PAK; 4 Internal Medicine, West Virginia University/Camden Clark Medical Center, Parkersburg, USA; 5 Internal Medicine, Western Michigan University Homer Stryker M.D. School of Medicine, Kalamazoo, USA; 6 Internal Medicine, Icahn School of Medicine at Mount Sinai, New York, USA; 7 Pathology and Laboratory Medicine, Mount Sinai Morningside, New York, USA

**Keywords:** attitude, belief, covid-19, meta-analysis, vaccine

## Abstract

The availability of an effective vaccine does not equate to its use; its effectiveness primarily depends on vaccine acceptance by the targeted population. Despite the rapid development and widespread access to the COVID-19 vaccine, herd immunity is yet to be achieved, with vaccine hesitancy as a major barrier. This study sought to systematically assess the beliefs, attitudes, and acceptance towards COVID-19 vaccines, including factors contributing to vaccination hesitancy in the Eastern and Western Hemispheres. A comprehensive search of articles was conducted through Scopus, PubMed, Embase, CINAHL, Cochrane CENTRAL, and Web of Science databases for studies published from inception to May 2023 using the Preferred Reporting Items for Systematic Reviews and Meta-Analyses (PRISMA) guidelines. Our search yielded 1154, of which 21 were eligible for inclusion. The rate of willingness or intention to vaccinate varied with the geographic region, from 12% in the United States to 93.9% in China. Four studies from the Western region and two from the Eastern region reported a low acceptance rate (defined as <50%): United States (12%), Spain (48.3%), Switzerland (38.6%), Europe (multi-national, 31%), Nepal (38.3%), and Oman (43%). Overall, vaccine acceptance was low to moderate in the general population and healthcare workers (HCWs) in both Eastern and Western Hemispheres except for China which reported high acceptance (defined as >75%) among the general population and HCWs. Demographic characteristics (female, younger age, and higher education) and non-demographic factors (knowledge about the COVID-19 vaccine and its development, history of influenza vaccination, perceived susceptibility or severity of infection, and the belief that vaccines are effective in controlling the pandemic) were associated with high acceptance rates or intentions to take the COVID-19 vaccine. On the other hand, mistrust of the vaccine, its safety and effectiveness, disinformation or poor awareness of the vaccine, side effect concerns, belief in natural immunity, previous adverse experience with the vaccines, and distrust in the information sources about the COVID-19 pandemic were associated with vaccination hesitancy. For better acceptance, COVID-19 vaccination campaign strategies should be modeled based on regional political, economic, and social contexts.

## Introduction and background

The COVID-19 pandemic is one of the most significant pandemics of the 21st century accounting for over 767 million infections and nearly seven million deaths as of July 2023 [1]. Due to its severity, pandemic control measures such as social distancing were prioritized alongside developing novel vaccines and pharmaceutic and biologic agents to treat patients with severe symptoms [2]. There is a global consensus that vaccination is the imminent and ultimate key to controlling the COVID-19 pandemic. For instance, in May 2020, the 73rd World Health Assembly issued a resolution that attributed comprehensive immunization as a global fundamental public health strategy to prevent, contain, and stop the spread of COVID-19 [3].

In response to the call, multiple COVID-19 vaccines were developed and tested across diverse populations, signifying a significant breakthrough in vaccine development to contain the pandemic. The US Food and Drug Administration issued the first emergency use authorization for the BTN162b2 vaccine (Pfizer-BioNTech) on December 11, 2020, to inoculate young adults aged 16 years and above [4]. Soon after, emergency use authorization was granted for mRNA-1273 (Moderna), AZD1222 (Oxford/AstraZeneca), and Ad26.COV2.5 (Janssen) vaccines [5]. By the first quarter of 2021, there were 85 vaccine candidates in the clinical trial phase and 184 in the pre-clinical phase [6].

However, reluctance to accept a potential vaccine was reported even before the widespread availability of COVID-19 vaccines in the general population and healthcare workers (HCWs) [7,8]. COVID-19 vaccine hesitancy has been a barrier to scaling up the vaccination rate for achieving herd immunity [9]. Vaccine hesitancy is a multi-factored phenomenon rooted in several factors such as an individual's socio-political stand, religious convictions, trust in government or the healthcare system, health/vaccine literacy, the source of information, and the perceived severity or risk of contracting the disease [10]. These factors tend to aggregate with other vaccine-specific factors, and as a result, the underlying cluster of factors of vaccine hesitancy varies by the vaccine. In the case of COVID-19, the rapid development and regulatory approval of the vaccine, political affiliations, and widespread misinformation about vaccine safety and efficacy and disease severity have played a key role in low public confidence in the vaccine [11,12]. As a result, significant disparities in regional vaccination rates have emerged. As of June 26, 2023, persons vaccinated with a complete primary series per 100 population stood at 31.5 for Africa, 51.3 for Eastern Mediterranean, 64.6 for Europe, 69.2 for Southeast Asia, 71.2 for the Americas, and 85.4 for Western Pacific regions [1].

Given the disparities in vaccination rates, region-specific interventions may be required to improve the willingness to vaccinate. Although several studies have explored the factors associated with COVID-19 at the country level, regional studies are scarce. Therefore, the present study sought to conduct a systematic review of the current works of literature regarding the facets of vaccine hesitancy, particularly the factors associated with the beliefs, attitudes, and acceptance of vaccines in the Western and Eastern Hemispheres.

## Review

Materials and methods

A rapid systematic review approach was utilized in conformity with the guidelines of the Preferred Reporting Items for Systematic Reviews and Meta-Analyses (PRISMA) reporting statement [13] to give valid and timely evidence that informs various decision-making concerning COVID-19 vaccination strategies.

Search Strategy

A systematic literature search was conducted for relevant articles published from inception to May 2023 in the following databases with customized retrieval strategies for each database: Scopus (Medline), PubMed, Embase, CINAHL, Cochrane CENTRAL, and Web of Science. Regarding the search syntax, the controlled vocabulary involved generic free-text search terms such as "population", "willingness", "attitude", "beliefs", and "vaccine hesitancy". The Medical Subject Headings (MeSH) vocabulary terms also included "vaccine", "COVID-19 vaccines", "sars-cov-2 vaccine", "vaccination refusal", "2019 novel coronavirus", and "coronavirus". The English synonyms were also used systematically as search syntax items in the databases to minimize the chances of missing relevant studies. In addition, the lists of bibliographies of the eligible articles and Google Scholar were manually searched to identify any potential reference for inclusion. This article was previously posted to the medRxiv preprint server on April 27, 2020.

Inclusion and Exclusion Criteria

Studies involving the general public and/or HCWs were included, where HCWs were defined as persons employed or studying within healthcare facilities, including physicians, medical students, and ancillary staff. Only published primary studies with structured designs, such as cross-sectional studies and semi-structured surveys, were considered. Articles that examined attitudes, acceptance, or beliefs towards COVID-19 vaccination, along with related factors, were included, with vaccination acceptance defined as the percentage of the population willing to accept vaccination when available. English-language articles were prioritized for inclusion, though relevant articles in other languages could be translated into English. Studies such as duplicates, commentaries, case reports, protocols, editorials, letters, conference abstracts, retracted articles, and articles in preprint were excluded, as were articles not available in full online or from low-quality sources.

Article Screening and Selection 

All the articles from the search were imported into the EndNote software (Clarivate, London, United Kingdom) [14] and de-duplicated. Titles and abstracts of the remaining articles were assessed, and those not meeting the inclusion criteria were excluded. The remaining articles were subjected to full-text screening. If more than one article utilized the same dataset, the one with the largest sample size was included. 

Two reviewers (F.S. and R.K.) conducted article screening and selection. Any differences between the reviewers were resolved through discussion. However, if the two reviewers could not arrive at a consensus on excluding or including some articles, the senior reviewer (G.H.) was consulted.

Data Extraction and Management

Abstraction of all the relevant data was simultaneously performed by two reviewers (F.S. and R.K.) independently using a standardized data extraction form and then exchanged for validation to prevent data inconsistency and errors. Any disagreement was resolved through discussion with the third reviewer (G.H.). Information that reflected the attitude or beliefs of the participants towards receiving COVID-19 vaccines were sought through either of the following measurements: rate of COVID-19 vaccine acceptance or rejection, rate of positive/negative response to specific vaccine uptake, and rate of individuals willing to enroll in a COVID-19 vaccine trial. The other relevant significant information that was sought included the following: the first author and publication year, the geographical context of the participants (i.e., country/city/setting), study design, survey period, study population of interest and sample sizes, data collection methods, demographic characteristics, and motivation and barriers towards vaccine uptake (vaccine hesitancy factors).

The vaccine acceptance rate was defined as the proportion of the population who were willing to or had taken the locally available COVID-19 vaccine and was categorized as low (<50%), moderate (50-75%), and high (>75%).

Evidence Quality Assessment and Risk of Bias

The selected articles' methodological quality and risk of bias were assessed using the Newcastle-Ottawa (NOS) quality scale independently by two reviewers (A.B.L. and S.C.). Any uncertainties were resolved by consulting the third reviewer (Y.M.N.). The NOS scale was developed to assess the quality of non-randomized studies of their design, content, and ease of use. The NOS has seven quality scoring systems under three main themes: selection, comparability, and outcome.

Results

Search Results and Study Selection

**Figure 1:**
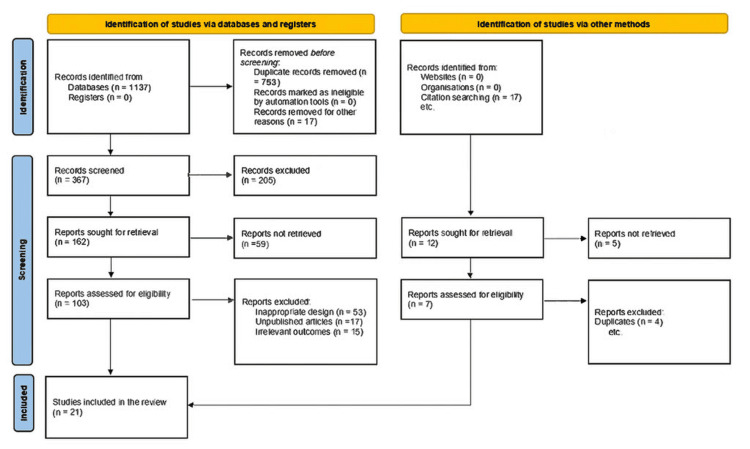
PRISMA flowchart for study screening and selection process PRISMA: Preferred Reporting Items for Systematic Reviews and Meta-Analyses

Our database search returned 1,137 articles, with an additional 17 from manual reference list screening. Seven hundred seventy-eight articles were removed during de-duplication. Titles and abstracts of the remaining 367 studies were screened, of which 103 articles were eligible for full-text screening. Finally, 21 articles fully meeting the inclusion criteria were identified and included in this systematic review. The flow of study screening and selection is shown in Figure 1.

Study Characteristics

The studies included in this review were published between 2020 and 2022, although most were published in 2021 (n=16). All the included studies adopted a cross-sectional design and specifically targeted adults aged above 18 years. The sample sizes ranged from 102 to 12,034 subjects.

Ten of the 21 studies were from the Eastern Hemisphere encompassing India [15,16], China [17,18], Indonesia [19], Nepal [20], Oman [21], Saudi Arabia [22,23], and the United Arab Emirates [24]. Studies from the Western Hemisphere originated from Spain [25], Switzerland [26], Poland [27], Slovakia [28], the United States [29-32], Canada [33], and Ecuador [34]. There was one European multi-national study [35].

The detailed characteristics of the included studies are presented in Table 1.

**Table 1:** Summary of the included studies §All studies were cross-sectional and conducted using questionnaire-based surveys. Age data are presented as mean±SD or median (IQR) whenever available. GP: general population; HCW: healthcare worker; SD: standard deviation; IQR: interquartile range

Region and author	Survey characteristics^§^			Vaccine acceptance rate	Predictors of COVID-19 vaccine uptake	Predictors of COVID-19 vaccine hesitancy
	Period	Modality	Population			
Tamil Nadu, India [15]	Jan 2021	Physical	564 GP participants ≥18 years	Not reported	Trust in the effectiveness of the vaccines	Mistrust in the health system or vaccines. Preference for natural immunity compared to vaccines
Chandigarh, India [16]	Jan 2021	Online	403 HCW participants ≥18 years	54.6%	Perceived susceptibility or severity of infections. Not concerned about the efficacy of new vaccines	Concerns about vaccine safety in terms of quality control and side effects. Doubt on efficacy
Eastern China [17]	Jun-Sept 2021	Online	2,158 cancer patients ≥18 years	75.95%	Alcohol consumption vs. non-drinkers. Income impacted by the pandemic. Knowledge vaccine development. Believing in the safety of the vaccine. Willingness to pay or recommend families and friends to get vaccinated	Being retired or unemployed. Undergoing multiple cancer therapies. Concerns that the vaccine might deteriorate the prognosis of cancer
China (national sample) [18]	Jan-Feb 2021	Online	1,779 HCW participants ≥18 years	93.9%	High or medium knowledge level about COVID-19 vaccines	Demographics: being female or a nurse, college degree. Low level of COVID-19 vaccine knowledge. Non-exposure to COVID-19
Bali, Indonesia [19]	Sep-Oct 2020	Online	779 GP participants aged 24 (20-26) years	60.8%	Conspiracy beliefs, trust in conventional media and authoritative sources of information	Not reported
Pokhara, Nepal [20]	Jan-Feb 2021	Physical and online	266 HCW participants ≥18 years	38.3%	Demographics: being male, high income or education, designation (managerial and admin. staffs). Prior SARS-CoV-2 diagnosis. Perception about the severity of the pandemic	Concern about vaccine safety
Oman (national sample) [21]	Dec 2020	Online	608 HCW participants ≥20 years	43%	Demographics: being male. Perceived adverse events (less) after vaccination. Positive attitude towards the vaccine. Trust in the government. Having sound knowledge of the COVID-19 vaccine	Previous experience with SARS-CoV-2
Saudi Arabia (national sample) [22]	Jan 2021	Online	758 GP participants ≥18 years	64%	Trust in the source of health information about COVID-19. Perception towards whether the vaccine is effective on emerging variants. Previous uptake of the influenza vaccine. Potential mandatory vaccination for international travel	Not reported
Saudi Arabia (national sample) [23]	Jun-Jul 2021	Online	581 GP participants ≥18 years	63.9%	Adequate information about the COVID-19 vaccine	Poor awareness about the vaccine's effectiveness
United Arab Emirates (national sample) [24]	Sep 2020	Online	2,705 GP participants ≥18 years	60.1%	Demographics: male gender, race (non-Emiratis), younger age group, and low educational attainment. Perceived increased personal or public risk of contracting the disease. Increased perception of serious outcomes from the disease	Unemployment. No history of recent vaccination. Doubting the severity of the situation or the vaccine's ability to control the pandemic. Mistrust in information from public authorities and their abilities to handle the pandemic
Spain (national sample) [25]	Nov-Dec 2020	Online	2,501 GP participants aged 40.2±13.6 years	48.3%	Demographics: male gender, older than 60, married, retired, high-level education. Health status: hypertension, immunodepression, hypercholesterolemia, respiratory disease, or overweight	Disinformation. Lack of political consensus
Switzerland (national sample) [26]	Jun-Jul 2020	Online	1,551 pregnant or breastfeeding women ≥18 years	Pregnant=29.7%; breastfeeding=38.6%	Demographics: age older than 40 years and higher educational level. History of influenza vaccination within the previous year. Obstetricians as the primary healthcare practitioner. Being in their third trimester of pregnancy	Not reported
Łódź, Poland [27]	Dec 2020-Jan 2021	Online	2,300 HCW participants. Age: HCW=31.4±8.4; control=26.9±9.05	HCW=82.95%; control=54.31%	Positive medical history of recommended vaccinations. Fear of catching SARS-CoV-2. Fear of passing on the disease to relatives	Development of long-term side effects after getting the vaccine. Depression
Northern Slovakia [28]	Aug-Sept 2021	Online	582 physicians, 542 non-physician HCW, 153 non-HCW aged 48.3±12.6 years	84.3%	Physician job type. History of SARS-CoV-2 or influenza vaccination. Compulsory vaccination for HCWs and compulsory vaccination for selected groups	Mistrust in the efficacy of the vaccine. Concerns of safety and side effects of the vaccines. Contraindications or expect a complicated vaccination course
Central/Eastern Europe [35]	Apr-May 2020	Online	623 HCW participants (304, 86, 90, and 143 from Croatia, Slovenia, Serbia, and Poland, respectively). Average age=37.6 years	31%	Demographics: age (adults) and educational level (higher). Knowledge about the pandemic	Not reported
United States (national sample) [29]	Apr-May 2020	Online	486 GP participants with multiple sclerosis ≥18 years	66%	Demographics: higher education levels. Higher perception of infection susceptibility. Perceived trustworthiness of information sources	Not reported
Philadelphia, USA [30]	Nov-Dec 2020	Online	12,034 HCW participants ≥18 years	63.7%	Protecting themselves, their family, or the community. Belief that vaccination is the best measure to prevent becoming seriously ill from SARS-CoV-2. Personal health status/vaccination history. Exposure to SARS-CoV-2	Concern about side effects/getting infected from the vaccine. Vaccines being too new; less information/not knowing enough about the vaccine. Mistrust/concerns about the vaccine not working
New York, USA [31]	Dec 2020-Jan 2021	Physical	102 participants from the Haredi‑Orthodox Jewish community aged 32 (25-38) years	12%	Not reported	Belief that natural infection is better than vaccination for developing immunity. Agreement that prior infection provides a path towards resuming communal life. Pandemic-related loss of trust in physicians
United States (national sample) [32]	Apr 2020	Physical and online	991 GP participants ≥18 years	57.6%	Demographics: younger age, Black race, and lower educational attainment. Not having received the influenza vaccine in the prior year	Vaccine-specific concerns. The need for more information. Anti-vaccine attitudes or beliefs. Lack of trust
Quebec, Canada [33]	Mar-Dec 2020	Online	6,037 GP participants ≥18 years	76% and 66% in the first and second waves	Demographics: male gender, older age (60 years of age and over), university education. Chronic medical status/conditions (having or living with someone with chronic medical conditions). Increased risk perceptions of COVID-19	Not reported
Azuay Province, Ecuador [34]	Feb 2021	Online	1,219 GP participants aged 32±13 years	91%	Demographics: older age, higher education levels (postgraduate education). History of a negative COVID-19 test. Higher perception of infection susceptibility. Belief that COVID-19 infection can be prevented with a vaccine	Not reported

Quality Appraisal and Assessment 

The results of the quality appraisal for the included studies are presented in Table 2. Fifteen studies scored 6, while the rest scored 5 on the NOS quality scale, indicating good quality.

**Table 2:** The Newcastle-Ottawa scale scores for quality appraisal

Study	Selection				Comparability		Outcome	Total score
	Representativeness of the exposed cohort	Selection of non-exposed/non-respondents	Ascertainments of exposure	Demonstration that outcome of interest was not present at the start of the study	Based on design	Based on analysis	Assessment of outcome; statistical test	
[15]	1	0	1	1	1	1	1	6
[16]	1	0	1	1	1	0	1	5
[17]	1	0	1	1	1	1	1	6
[18]	1	0	1	1	1	1	1	6
[19]	1	0	1	1	1	1	1	6
[20]	1	0	1	1	1	1	1	6
[21]	1	0	1	1	1	1	1	6
[22]	1	0	1	1	1	1	1	6
[23]	1	0	1	1	1	1	1	6
[24]	1	0	1	1	1	0	1	5
[25]	1	0	1	1	1	1	1	6
[26]	1	0	1	1	1	1	1	6
[27]	1	0	1	1	1	0	1	5
[28]	1	0	1	1	1	1	1	6
[29]	1	0	1	1	1	0	1	5
[30]	1	0	1	1	1	1	1	6
[31]	1	0	1	1	1	0	1	5
[32]	1	0	1	1	1	0	1	5
[33]	1	0	1	1	1	1	1	6
[34]	1	0	1	1	1	1	1	6
[35]	1	0	1	1	1	1	1	6

Discussion

*Summary of Key Findings* 

A total of 21 studies were identified from 14 countries in the Eastern and Western Hemispheres that explored factors associated with attitudes, beliefs, and intentions to accept the COVID-19 vaccine. Findings indicated that COVID-19 vaccine acceptance was low to moderate in both the general population and HCWs. Demographic characteristics, including age, gender, education, and social status, were strongly associated with the willingness to vaccinate against COVID-19. Additionally, non-demographic factors such as sound knowledge about the COVID-19 vaccine and its development, history of previous influenza vaccination, perceived risks (including fear of contracting the family, protecting self, family, or community), the trustworthiness of the information source regarding COVID-19 disease, and the beliefs that the virus can be prevented using the vaccine or a positive attitude towards the vaccine and its efficacy were associated with high acceptance rates or intentions to take the COVID-19 vaccine. On the other hand, mistrust of the vaccine, its safety and effectiveness, disinformation or poor awareness of the vaccine, side effect concerns, belief in natural immunity, previous adverse experience with the vaccines, and distrust in the information sources about the COVID-19 pandemic were associated with vaccination hesitancy.

Acceptance Rates

All but one article [15] evaluated the willingness to receive the COVID-19 vaccine. The rate of willingness or intention to vaccinate varied with the geographic region from 12% in New York, USA [31], to 93.9% in China [18]. Four studies from the Western region and two from the Eastern region reported a low acceptance rate: United States (12%) [31], Spain (48.3%) [25], Switzerland (38.6%) [26], Europe (multi-national, 31%) [35], Nepal (38.3%) [20], and Oman (43%) [21].

Demographic Factors Associated With Attitudes/Beliefs/Intentions to Accept the COVID-19 Vaccine

The factors associated with the beliefs, attitudes, and intentions towards COVID-19 vaccine acceptance were analyzed in 11 of the included studies [20,21,24-26,28,29,32-35], which used either or both multivariate and univariate logistical regression analysis to account for the confounding variables. The studies noted several demographic factors associated with COVID-19 vaccine uptake, including age, gender, education level, occupation or social status, and race. Five studies reported that the male sex strongly predicted acceptance or higher intentions to vaccinate against COVID-19 than the female gender [20,21,24,25,33]. Of the seven studies that analyzed age, five studies [20,21,33-35] indicated that being above 40 years strongly correlated with vaccine acceptance, while the other two studies showed that younger age [24,32] predicted the intention to take COVID-19 vaccine among HCW and the general population. Six studies indicated that higher education levels [20,25,29,33-35], especially degree attainment, strongly predicted higher intention towards COVID-19 vaccine acceptance, contrary to two studies that showed that low education attainment [24,32] was associated with a high likelihood of vaccination acceptance. Regarding social status, studies indicated factors such as marriage, retirement, and high income as determinants of COVID-19 vaccine acceptance [25]. Nevertheless, two studies reported that identifying as White was associated with a greater intention to vaccinate than those identifying as Black, Asian, Hispanic, or others [30,32]. Among HCWs, designation or the type of work was a strong predictor of vaccination [20,28].

Non-demographic Factors Associated With Attitudes/Beliefs/Intentions to Accept the COVID-19 Vaccine

The included studies also reported factors unrelated to population demographics that correlated with the uptake of the COVID-19 vaccine. In particular, the high-risk perceptions towards COVID-19, including fear of contracting the disease and perception towards protecting self, relatives, family, and community, were identified by eight studies as a significant contributor to acceptance and intention to take COVID-19 vaccine [16,20,24,27,29,30,33,34].

Furthermore, five studies weighed the belief that COVID-19 disease is preventable through vaccination, a low risk of adverse effects, or a positive attitude towards the vaccine [15,19,21,30,34] as crucial contributors to vaccination acceptance. Four other studies identified sound knowledge of the COVID-19 pandemic and vaccine development as important factors for accepting COVID-19 vaccination [18,21,23,25]. Five studies also found that previous influenza vaccination correlated to the likelihood of vaccination against COVID-19 [22,26-28,32]. Trustworthiness of the health information source about COVID-19, including media, authorities, and the government, also emanated as a strong predictor in four included studies. The other crucial factors associated with beliefs, attitudes, or intentions to take the COVID-19 vaccine included health statuses such as chronic disease conditions, beliefs in conspiracy theories, willingness to recommend, COVID-19 exposure or previous diagnosis with COVID-19, and mandatory COVID-19 vaccination orders.

Reasons for COVID-19 Vaccine Hesitancy (Non-acceptance)

Fifteen articles identified factors associated with COVID-19 vaccine hesitancy. Eight studies identified the fear of developing severe side effects after vaccination [16,17,20,24,27,28,30,32]. Most of the individuals who were skeptical of developing side effects considered the COVID-19 vaccines unsafe and thus rejected them. Individuals also failed to accept the vaccines due to mistrust; rather, vaccination hesitancy was prompted since the targeted population showed distrust in the efficacy or effectiveness of the vaccines to treat or minimize the COVID-19 pandemic.

Four studies highlighted disinformation, lack of or poor awareness of the COVID-19 vaccine, and its efficacy as barriers to vaccine acceptance [18,23,25,30]. Another critical variable was distrust in COVID-19 information sources, which included pandemic-related loss of trust in physicians, the health system, and the authorities [15,24,32]. Three studies also associated the belief in natural immunity, or a belief in the perception that natural infection is better than vaccination for developing immunity, as well as the anti-vaccine beliefs and attitudes to strongly predict vaccine hesitancy [15,31,32]. Other factors associated with vaccine hesitancy included the vaccine being new, previous adverse effects or development of long-term side effects following COVID-19 vaccination, non-exposure to COVID-19, lacking a recent vaccination history, and doubts about the severity of the COVID-19 pandemic. One study identified demographic factors, such as female, retired, or unemployed, as factors associated with non-acceptance of the COVID-19 vaccine [18].

Regional Variations in Vaccine Acceptance

In the Eastern region, vaccine acceptance varied from low in Nepal and Oman to moderate in India, Indonesia, Saudi Arabia, and the United Arab Emirates. However, two studies from China reported high vaccine acceptance rates. Consistent with our findings, a previous systematic review by Sallam [36] found that vaccine acceptance in Southeast Asia was relatively high (90%) compared to Middle Eastern countries (below 30%). Al-Jayyousi et al. [37] attributed the low acceptance of the COVID-19 vaccine to the widespread beliefs in conspiracy theories that negatively impacted vaccine uptake. Sallam [36] also cited low trust in the government and low education levels in Middle Eastern countries, resulting in a low willingness to vaccinate. COVID-19 vaccine hesitancy has been reported in 6.3-56.2% of the population in South Asian countries, with safety and efficacy concerns as predominant factors [38].

However, it is noteworthy that the two studies from China exclusively involved HCWs [18] and cancer patients [17]. Contrary to our expectations, HCWs did not demonstrate a consistently higher acceptance rate than the general population across the region. For instance, Paudel et al. [20] sampled 266 Nepalese HCWs with an acceptance rate of 38.3%, Awaidy et al. [21] sampled 608 Omani HCWs with an acceptance rate of 43%, and Jose et al. [16] sampled 403 Indian HCWs with an acceptance rate of 54.6%, while Li et al. [18] reported a vaccine acceptance rate of 93.9% in a nationally representative sample of Chinese HCWs. These variations may be attributed to the national mandate to vaccinate HCWs in some countries [39]. For instance, vaccination against COVID-19 has been compulsory for all people employed at hospitals in China since the first quarter of 2021 [40], while countries like India prioritized but did not mandate the vaccination of HCWs [41].

In the Western region, low vaccination acceptance was reported from European countries [25-27]. In contrast, one study from Canada [33] and two from the United States [29,32] reported a moderate vaccine acceptance rate among the general population. While another study from the United States reported a very low acceptance rate of 12% [31], the participants were derived from a single community (Haredi‑Orthodox Jewish community). Our search strategy yielded only one article from South America (Ecuador), which reported a high vaccine acceptance rate in the general population. Although low high vaccine acceptance rates were reported among HCWs in Europe in the first quarter of 2020, later surveys reported high vaccine acceptance rates [27,28]. Vaccine acceptance among HCWs in the United States remained moderate in late 2020 [30], on par with the general population. 

Consistent with our findings, a recent meta-analysis reported 61% vaccine acceptance among general populations and 55% among HCWs in the United States [42]. However, in the United States, low vaccine acceptance may not be solely related to vaccine hesitancy. Instead, they may be driven by the syngenetic effects of several factors such as gender (women), race (Black and Hispanic), age (younger adults), political affiliations (Republican), low education and income, and residing in rural areas [42,43]. Similarly, moderate vaccine acceptance rates have been reported in European countries in both the general population and HCWs, with low trust/confidence in the vaccines, government and medical system, and health/vaccine literacy as the main drivers of vaccine hesitancy [44,45]. Even in the early phase of vaccine rollout, Asian countries like China and South Korea reported higher vaccine acceptance and greater trust in government than the United States, the United Kingdom, and European countries [46,47].

Concerns regarding the efficacy, safety, and effectiveness of the COVID-19 vaccines have been major factors for vaccine hesitancy, while perceived susceptibility and severity of COVID-19, trust in the government, and demographic factors such as female gender, younger age, higher educational attainment, and high income have been associated with willingness to vaccinate throughout the pandemic [46-54]. These vaccine refusal and acceptance predictors may be directly related to COVID-19-related disinformation. As Al-Amer et al. [39] noted, myths, rumors, and false beliefs on vaccines peddled by anti-vaccine individuals and media, especially social media, threatened vaccination uptake. Similarly, Jin et al. [55] supported that the beliefs in conspiracy theories circulated in the media by vaccine-averse individuals regarding the vaccine's side effects significantly influenced people's decision to vaccinate. For instance, Reno et al. [56] showed that males were less likely to believe in conspiracy theories and perceived greater severity of COVID-19 disease and had a higher willingness to vaccinate, while Troiano and Nardi [57] demonstrated greater concern towards the safety of the vaccines and distrusted the quality and impartiality of the vaccine information among females with a negative effect on vaccine acceptance. Physician policymakers and public health practitioners must consider the complex social dynamics in politically divided countries, where certain groups may blame others for promoting vaccines with negative intentions. Recognizing these dynamics is crucial for designing and implementing effective and culturally sensitive vaccination campaigns that build public trust and combat misinformation. Moreover, vaccine hesitancy could also result from underestimating the risk and severity of infection. The history of influenza vaccination was another critical facilitator of vaccine acceptance attitude, consistent with other studies [7,58].

Strengths and limitations

This systematic review provides updated evidence on the beliefs, attitudes, and acceptance of COVID-19 in both HCWs and non-HCWs in the Eastern and Western regions of the world, which can assist in developing region-specific policies to mitigate potential outbreaks of severe COVID-19 variants or other infectious diseases. Nonetheless, this study has several limitations that limit the generalizability of comprehensiveness of the findings. First, we only included peer-reviewed published articles with the potential risk of missing more recent data that may have been available in the grey literature and preprints. Second, all included studies were conducted online or through network surveys, which could compromise participant selection, especially with low participant turnout or response rate. Moreover, participation was voluntary in most studies, increasing the risk of selection bias. Third, all included articles were cross-sectional studies, which are typically descriptive, thereby limiting the scope of drawing comprehensive causal inferences. Besides, cross-sectional studies are limited to a particular point in time. Inevitably, people's attitudes, willingness, and beliefs might have changed over time to influence the acceptance of vaccines.

## Conclusions

Most countries in the Eastern and Western regions exhibited low to moderate vaccine acceptance rates in the general population and HCWs. Demographic characteristics such as male gender, older age, higher education attainment, and having high socioeconomic status strongly related to the positive attitude towards intentions to vaccinate. Other positive predictive factors included self-perceived risks, including fear of contracting the virus, previous history of influenza vaccination, sound knowledge of the vaccine, positive attitude towards the vaccine and its efficacy, trustworthiness of information sources, and mandatory vaccination orders. On the other hand, safety, efficacy, and effectiveness concerns, distrust of the authority or government and information sources, belief in natural immunity, and disinformation were barriers that led to vaccine hesitancy. Therefore, COVID-19 vaccination campaign strategies should be modeled based on regional political, economic, and social contexts for better acceptance.

## References

[1] (2023). WHO COVID-19 dashboard. https://covid19.who.int/.

[2] Liu C, Zhou Q, Li Y (2020). Research and development on therapeutic agents and vaccines for COVID-19 and related human coronavirus diseases. ACS Cent Sci.

[3] (2020). Seventy-third World Health Assembly. Geneva.

[4] Oliver SE, Gargano JW, Marin M (2020). The Advisory Committee on Immunization Practices' interim recommendation for use of Pfizer-BioNTech COVID-19 vaccine - United States, December 2020. MMWR Morb Mortal Wkly Rep.

[5] Kalinke U, Barouch DH, Rizzi R, Lagkadinou E, Türeci Ö, Pather S, Neels P (2022). Clinical development and approval of COVID-19 vaccines. Expert Rev Vaccines.

[6] Ahmed S, Khan S, Imran I (2021). Vaccine development against COVID-19: study from pre-clinical phases to clinical trials and global use. Vaccines (Basel).

[7] Dror AA, Eisenbach N, Taiber S (2020). Vaccine hesitancy: the next challenge in the fight against COVID-19. Eur J Epidemiol.

[8] Roberts HA, Clark DA, Kalina C, Sherman C, Brislin S, Heitzeg MM, Hicks BM (2022). To vax or not to vax: predictors of anti-vax attitudes and COVID-19 vaccine hesitancy prior to widespread vaccine availability. PLoS One.

[9] Gerretsen P, Kim J, Quilty L (2021). Vaccine hesitancy is a barrier to achieving equitable herd immunity among racial minorities. Front Med (Lausanne).

[10] Dubé E, Laberge C, Guay M, Bramadat P, Roy R, Bettinger J (2013). Vaccine hesitancy: an overview. Hum Vaccin Immunother.

[11] Hudson A, Montelpare WJ (2021). Predictors of vaccine hesitancy: implications for COVID-19 public health messaging. Int J Environ Res Public Health.

[12] Sabahelzain MM, Hartigan-Go K, Larson HJ (2021). The politics of Covid-19 vaccine confidence. Curr Opin Immunol.

[13] Page MJ, McKenzie JE, Bossuyt PM (2021). The PRISMA 2020 statement: an updated guideline for reporting systematic reviews. BMJ.

[14] (2013). EndNote. https://support.clarivate.com/Endnote/s/article/Citing-the-EndNote-program-as-a-reference?language=en_US.

[15] Danabal KG, Magesh SS, Saravanan S, Gopichandran V (2021). Attitude towards COVID 19 vaccines and vaccine hesitancy in urban and rural communities in Tamil Nadu, India - a community based survey. BMC Health Serv Res.

[16] Jose S, Cyriac MC, Dhandapani M, Joseph J (2022). COVID-19 vaccination intention and hesitancy: mistrust on COVID-19 vaccine benefit a major driver for vaccine hesitancy among healthcare workers; a cross-sectional study in North India. J Prev Med Hyg.

[17] Hong J, Xu XW, Yang J (2022). Knowledge about, attitude and acceptance towards, and predictors of intention to receive the COVID-19 vaccine among cancer patients in Eastern China: a cross-sectional survey. J Integr Med.

[18] Li XH, Chen L, Pan QN (2021). Vaccination status, acceptance, and knowledge toward a COVID-19 vaccine among healthcare workers: a cross-sectional survey in China. Hum Vaccin Immunother.

[19] Wirawan GB, Mahardani PN, Cahyani MR, Laksmi NL, Januraga PP (2021). Conspiracy beliefs and trust as determinants of COVID-19 vaccine acceptance in Bali, Indonesia: cross-sectional study. Pers Individ Dif.

[20] Paudel S, Palaian S, Shankar PR, Subedi N (2021). Risk perception and hesitancy toward COVID-19 vaccination among healthcare workers and staff at a medical college in Nepal. Risk Manag Healthc Policy.

[21] Awaidy ST, Al Siyabi H, Khatiwada M (2022). Assessing COVID-19 vaccine's acceptability amongst health care workers in Oman: a cross-sectional study. J Infect Public Health.

[22] Alshahrani SM, Dehom S, Almutairi D (2021). Acceptability of COVID-19 vaccination in Saudi Arabia: a cross-sectional study using a web-based survey. Hum Vaccin Immunother.

[23] Samannodi M, Alwafi H, Naser AY (2021). Assessment of caregiver willingness to vaccinate their children against COVID-19 in Saudi Arabia: a cross-sectional study. Hum Vaccin Immunother.

[24] Albahri AH, Alnaqbi SA, Alshaali AO, Alnaqbi SA, Shahdoor SM (2021). COVID-19 vaccine acceptance in a sample from the United Arab Emirates general adult population: a cross-sectional survey, 2020. Front Public Health.

[25] Rodríguez-Blanco N, Montero-Navarro S, Botella-Rico JM, Felipe-Gómez AJ, Sánchez-Más J, Tuells J (2021). Willingness to be vaccinated against COVID-19 in Spain before the start of vaccination: a cross-sectional study. Int J Environ Res Public Health.

[26] Stuckelberger S, Favre G, Ceulemans M (2021). SARS-CoV-2 vaccine willingness among pregnant and breastfeeding women during the first pandemic wave: a cross-sectional study in Switzerland. Viruses.

[27] Szmyd B, Karuga FF, Bartoszek A (2021). Attitude and behaviors towards SARS-CoV-2 vaccination among healthcare workers: a cross-sectional study from Poland. Vaccines (Basel).

[28] Ulbrichtova R, Svihrova V, Tatarkova M, Hudeckova H, Svihra J (2021). Acceptance of COVID-19 vaccination among healthcare and non-healthcare workers of hospitals and outpatient clinics in the northern region of Slovakia. Int J Environ Res Public Health.

[29] Ehde DM, Roberts MK, Herring TE, Alschuler KN (2021). Willingness to obtain COVID-19 vaccination in adults with multiple sclerosis in the United States. Mult Scler Relat Disord.

[30] Kuter BJ, Browne S, Momplaisir FM (2021). Perspectives on the receipt of a COVID-19 vaccine: a survey of employees in two large hospitals in Philadelphia. Vaccine.

[31] Carmody ER, Zander D, Klein EJ, Mulligan MJ, Caplan AL (2021). Knowledge and attitudes toward Covid-19 and vaccines among a New York Haredi-Orthodox Jewish community. J Community Health.

[32] Fisher KA, Bloomstone SJ, Walder J, Crawford S, Fouayzi H, Mazor KM (2020). Attitudes toward a potential SARS-CoV-2 vaccine: a survey of U.S. adults. Ann Intern Med.

[33] Dubé È, Dionne M, Pelletier C, Hamel D, Gadio S (2021). COVID-19 vaccination attitudes and intention among Quebecers during the first and second waves of the pandemic: findings from repeated cross-sectional surveys. Hum Vaccin Immunother.

[34] Jaramillo-Monge J, Obimpeh M, Vega B, Acurio D, Boven A, Verhoeven V, Colebunders R (2021). COVID-19 vaccine acceptance in Azuay Province, Ecuador: a cross-sectional online survey. Vaccines (Basel).

[35] Kregar Velikonja N, Globevnik Velikonja V, Verdenik I, Jurišić I, Stanisavljević S, Dobrowolska B, Erjavec K (2022). Vaccination intention among healthcare workers during the first wave of the coronavirus disease 2019 pandemic in relation to knowledge: a cross-sectional study in Croatia, Slovenia, Serbia, and Poland. Croat Med J.

[36] Sallam M (2021). COVID-19 vaccine hesitancy worldwide: a concise systematic review of vaccine acceptance rates. Vaccines (Basel).

[37] Al-Jayyousi GF, Sherbash MA, Ali LA, El-Heneidy A, Alhussaini NW, Elhassan ME, Nazzal MA (2021). Factors influencing public attitudes towards COVID-19 vaccination: a scoping review informed by the socio-ecological model. Vaccines (Basel).

[38] Ennab F, Qasba RK, Uday U (2022). COVID-19 vaccine hesitancy: a narrative review of four South Asian countries. Front Public Health.

[39] Maneze D, Salamonson Y, Grollman M, Montayre J, Ramjan L (2023). Mandatory COVID-19 vaccination for healthcare workers: a discussion paper. Int J Nurs Stud.

[40] Cheng Y, Li T, Zheng Y, Xu B, Bi Y, Hu Y, Zhou YH (2022). Self-reported adverse events among Chinese healthcare workers immunized with COVID-19 vaccines composed of inactivated SARS-CoV-2. Hum Vaccin Immunother.

[41] Purohit N, Chugh Y, Bahuguna P, Prinja S (2022). COVID-19 management: the vaccination drive in India. Health Policy Technol.

[42] Dhanani LY, Franz B (2022). A meta-analysis of COVID-19 vaccine attitudes and demographic characteristics in the United States. Public Health.

[43] Hu S, Xiong C, Li Q, Wang Z, Jiang Y (2022). COVID-19 vaccine hesitancy cannot fully explain disparities in vaccination coverage across the contiguous United States. Vaccine.

[44] Popa AD, Enache AI, Popa IV, Antoniu SA, Dragomir RA, Burlacu A (2022). Determinants of the hesitancy toward COVID-19 vaccination in Eastern European countries and the relationship with health and vaccine literacy: a literature review. Vaccines (Basel).

[45] Heyerdahl LW, Vray M, Lana B (2022). Conditionality of COVID-19 vaccine acceptance in European countries. Vaccine.

[46] Lin C, Tu P, Beitsch LM (2020). Confidence and receptivity for COVID-19 vaccines: a rapid systematic review. Vaccines (Basel).

[47] Wang Q, Yang L, Jin H, Lin L (2021). Vaccination against COVID-19: a systematic review and meta-analysis of acceptability and its predictors. Prev Med.

[48] Kreps S, Prasad S, Brownstein JS, Hswen Y, Garibaldi BT, Zhang B, Kriner DL (2020). Factors associated with US adults' likelihood of accepting COVID-19 vaccination. JAMA Netw Open.

[49] Pogue K, Jensen JL, Stancil CK (2020). Influences on attitudes regarding potential COVID-19 vaccination in the United States. Vaccines (Basel).

[50] Schwarzinger M, Watson V, Arwidson P, Alla F, Luchini S (2021). COVID-19 vaccine hesitancy in a representative working-age population in France: a survey experiment based on vaccine characteristics. Lancet Public Health.

[51] Al-Amer R, Maneze D, Everett B, Montayre J, Villarosa AR, Dwekat E, Salamonson Y (2022). COVID-19 vaccination intention in the first year of the pandemic: a systematic review. J Clin Nurs.

[52] Miyachi T, Takita M, Senoo Y, Yamamoto K (2020). Lower trust in national government links to no history of vaccination. Lancet.

[53] Larson HJ, Clarke RM, Jarrett C, Eckersberger E, Levine Z, Schulz WS, Paterson P (2018). Measuring trust in vaccination: a systematic review. Hum Vaccin Immunother.

[54] Lee C, Whetten K, Omer S, Pan W, Salmon D (2016). Hurdles to herd immunity: distrust of government and vaccine refusal in the US, 2002-2003. Vaccine.

[55] Jin Q, Raza SH, Yousaf M, Zaman U, Siang JM (2021). Can communication strategies combat COVID-19 vaccine hesitancy with trade-off between public service messages and public skepticism? Experimental evidence from Pakistan. Vaccines (Basel).

[56] Reno C, Maietti E, Fantini MP, Savoia E, Manzoli L, Montalti M, Gori D (2021). Enhancing COVID-19 vaccines acceptance: results from a survey on vaccine hesitancy in Northern Italy. Vaccines (Basel).

[57] Troiano G, Nardi A (2021). Vaccine hesitancy in the era of COVID-19. Public Health.

[58] Reiter PL, Pennell ML, Katz ML (2020). Acceptability of a COVID-19 vaccine among adults in the United States: how many people would get vaccinated?. Vaccine.

